# Cytotoxic *Escherichia coli* strains encoding colibactin isolated from immunocompromised mice with urosepsis and meningitis

**DOI:** 10.1371/journal.pone.0194443

**Published:** 2018-03-19

**Authors:** Vasudevan Bakthavatchalu, Katherine J. Wert, Yan Feng, Anthony Mannion, Zhongming Ge, Alexis Garcia, Kathleen E. Scott, Tyler J. Caron, Carolyn M. Madden, Johanne T. Jacobsen, Gabriel Victora, Rudolf Jaenisch, James G. Fox

**Affiliations:** 1 Division of Comparative Medicine, Massachusetts Institute of Technology, Cambridge, Massachusetts, United States of America; 2 Whitehead Institute for Biomedical Research, Cambridge, Massachusetts, United States of America; Instituto Butantan, BRAZIL

## Abstract

Immune-compromised mouse models allow for testing the preclinical efficacy of human cell transplantations and gene therapy strategies before moving forward to clinical trials. However, CRISPR/Cas9 gene editing of the *W*^*sh*^*/W*^*sh*^ mouse strain to create an immune-compromised model lacking function of *Rag2* and *Il2rγ* led to unexpected morbidity and mortality. This warranted an investigation to ascertain the cause and predisposing factors associated with the outbreak. Postmortem examination was performed on 15 moribund mice. The main lesions observed in these mice consisted of ascending urogenital tract infections, suppurative otitis media, pneumonia, myocarditis, and meningoencephalomyelitis. As *Escherichia coli* strains harboring polyketide synthase (*pks)* genomic island were recently isolated from laboratory mice, the tissue sections from the urogenital tract, heart, and middle ear were subjected to *E*. *coli* specific PNA-FISH assay that revealed discrete colonies of *E*. *coli* associated with the lesions. Microbiological examination and 16S rRNA sequencing confirmed *E*. coli-induced infection and septicemia in the affected mice. Further characterization by *clb* gene analysis and colibactin toxicity assays of the *pks+ E*. *coli* revealed colibactin-associated cytotoxicity. Rederivation of the transgenic mice using embryo transfer produced mice with an intestinal flora devoid of *pks+ E*. *coli*. Importantly, these barrier-maintained rederived mice have produced multiple litters without adverse health effects. This report is the first to describe acute morbidity and mortality associated with *pks+ E*. *coli* urosepsis and meningitis in immunocompromised mice, and highlights the importance of monitoring and exclusion of colibactin-producing *pks+ E*. *coli*.

## Introduction

Conversion of common murine models to an immune-compromised state has become highly desirable for translational research. The creation of immune-compromised mouse strains allows for the preclinical efficacy of human cell transplantations and gene therapy strategies to be tested in small rodent systems before moving forward to human clinical trials. Recently, we have utilized the *W*^*sh*^*/W*^*sh*^ mouse strain to examine human neural crest cell contribution during embryonic and post-natal mouse development [1]. In order to increase human cell incorporation into the *W*^*sh*^*/W*^*sh*^ mice, as well as provide a future system for human disease modeling, we used CRISPR (clustered regularly interspaced short palindromic repeat) and Cas (CRISPR-associated) proteins to knockout interleukin 2 receptor subunit gamma (*Il2rγ*) and recombination activating gene-2 (*Rag2*) in the *W*^*sh*^*/W*^*sh*^ mice. The conversion of the *W*^*sh*^*/W*^*sh*^ mice to an immune-compromised state led to unexpected morbidity and mortality. This warranted an investigation to ascertain the cause and predisposing factors associated with the outbreak.

*Escherichia coli (E*. *coli)* strains have been frequently isolated from rodents, but are not routinely or completely characterized; these bacteria are commonly considered commensals and are not currently excluded from specific pathogen-free mouse colonies. However, pathogenic *E*. *coli* can encode various virulence factors, which are classified into different subtypes, such as enteropathogenic *E*. *coli* (EPEC), enterohemorrhagic *E*. *coli* (EHEC), enterotoxigenic *E*. *coli* (ETEC), enteroinvasive *E*. *coli* (EIEC), enteroaggregative *E*. *coli* (EAggEC), diffusely adhering *E*. *coli* (DAEC) and adherent-invasive *E*. *coli* (AIEC) [2]. Another group called extraintestinal pathogenic *E*. *coli* (ExPEC) belongs to the B2 *E*. *coli* phylogroup and are associated with human cases of meningitis, septicemia, and urinary tract infections (ExPEC) [3,4].

The polyketide synthase (*pks)* is a 54-kb genomic island that encodes colibactin (Clb) gene cluster [5,6]. The genotoxic metabolite colibactin acts as a cyclomodulin that induces DNA damage and cell cycle arrest in mammalian cells. [5–9]. The *pks* genomic island is highly conserved in *Enterobacteriaceae* and has been isolated from commensal *E*. *coli* strains (B2 and Nissle 1917 *E*. *coli*), *Citrobacter koseri*, *Klebsiella pneumoniae*, and *Enterobacter aerogenes* [5,7]. A study identified that IL10^-/-^ mice with experimentally-induced chronic lower bowel inflammation were colonized with a specific *E*. *coli* (O7:H7:K1) strain of phylogenetic group B2 that encodes high number of virulence associated genes [10]. The tumor promoting effects of *pks+* NC101 *E*. *coli* strain (O2:H6/41) were identified in germfree IL10^-/-^ mice carcinogenicity studies Further, carcinogenicity studies in germfree IL10^-/-^ mice monoassociated with the *pks+* NC101 *E*. *coli* strain (O2:H6/41) identified its tumor promoting effects [11,12]. The *pks+* NC101 *E*. *coli* strain associated promotional events were specifically attributed to the *pks* genomic island and excluded the role of inflammation in the formation of invasive carcinoma [11]. IL10^-/-^ mice monoassociated with a murine *E*. *coli* strain from wildtype mice (later identified as NC101) induced typhlitis due to increased synthesis of interferon γ and IL4 by CD4^+^ T cells [13]. *pks+ E*. *coli* strains have also been utilized experimentally in rodent models to study urosepsis and septicemia [14,15].

We recently identified *pks+ E*. *coli* strains from the gastrointestinal tract of commercially available mice, as well as mice maintained in a large biomedical research institute, and demonstrated the cytotoxic effects of colibactin produced by the isolated *pks+ E*. *coli* in a cell culture system [16]. These *pks+ E*. *coli* strains, which colonize asymptomatic humans, are also associated with inflammation, septicemia, meningitis, and urinary tract infections [3,4], as well as being commonly isolated in colon cancer patients [17]. The purpose of this report is to describe acute morbidity and mortality in immunocompromised mice, from which colibactin producing *E*. *coli* was isolated from blood, genitourinary tract, and brain. As genome-engineering technologies have allowed for an increase in the efficiency and reduction in the time it takes to generate knockout mouse models, many murine models used in human research are becoming immune-compromised in order to test human cell transplantations and viral gene therapies for translational research. The abrupt increase in mortality and morbidity associated with *pks+ E*. *coli* in immune-compromised mice entails routine and complete characterization of these bacteria to ensure exclusion of colibactin-producing *pks+ E*. *coli* strains from specific pathogen-free mouse colonies.

## Materials and methods

### Ethics statement

All animal experiments were approved by the Massachusetts Institute of Technology Committee on Animal Care (Protocol #0916-058-19: Gene Disease, Cancer, and Mammalian Development) and all animal procedures were performed following the National Institute of Health guidelines. Our facility conforms to Federal guidelines and has a PHS Approved Animal Welfare Assurance (#A3125-01). Mice were monitored at least twice daily, and any adverse conditions (e.g. difficulty with ambulation, hunched posture, body condition score (BCS) < 2, ruffled fur) were immediately brought to the attention of the veterinary staff. The mice for this study displayed normal behavior and health until suddenly showing signs of morbidity, and would succumb within 24 hours. Mice that became moribund/severely ill during this study were euthanized with CO_2_ exposure followed by cervical dislocation, and tissues were collected for analysis.

### Production of Cas9 mRNA and sgRNA

Bicistronic expression vector px330 expressing Cas9 and sgRNA [18] was digested with BbsI and treated with Antarctic Phosphatase, and the linearized vector was gel purified. A pair of oligos (Table 1) for each targeting site was annealed, phosphorylated, and ligated to the linearized vector. T7 promoter was added to the Cas9 coding region by PCR amplification using primers Cas9 F and R (Table 1). The T7-Cas9 PCR product was gel purified and used as the template for *in vitro* transcription (IVT) using the mMESSAGE mMACHINE T7 ULTRA kit (Life Technologies). The T7 promoter was added to sgRNA templates by PCR amplification using the primers listed in Table 1 of a previously published paper by the Jaenisch Lab [19]. The T7-sgRNA PCR product was gel purified and used as the template for IVT using the MEGAshortscript T7 kit (Life Technologies). Both the Cas9 mRNA and the sgRNAs were purified using MEGAclear kit (Life Technologies) and eluted in RNase-free water.

**Table 1:** Oligonucleotides used in this study.

Gene	Oligonucleotide	Cloning Step
*Rag2*	CACCGTATTGTGGGTGGTTATCAGC	sgRNA
*Rag2*	CACCGCCCTCAGCAGGAGCAGCTGA	sgRNA
*Il2rγ*	AAACGCTGATAACCACCCACAATAC	sgRNA
*Il2rγ*	AAACTCAGCTGCTCCTGCTGAGGGC	sgRNA
*Rag2*	TTAATACGACTCACTATAGTATTGTGGGTGGTTATCAGC	*in vitro* Transcription
*Il2rγ*	TTAATACGACTCACTATAGCCCTCAGCAGGAGCAGCTGA	*in vitro* Transcription
*Rag2*	GGAAAAGCATGGGTGTTCTC	RFLP Assay
*Rag2*	TCCTGGTATGCCAAGGAAAA	RFLP Assay
*Il2rγ*	TCTCCCTGGGGACTTAGCTT	RFLP Assay
*Il2rγ*	AGGGGCAGAGTAGGAGCACT	RFLP Assay

### One-cell embryo injection

Mice were obtained from the Jackson Laboratory and maintained in the Whitehead Institute animal facility. *W*^*sh*^*/W*^*sh*^ female mice on a C57BL/6 background and ICR mice were used as embryo donors and foster mothers, respectively. Superovulated female *W*^*sh*^*/W*^*sh*^ mice (7–8 weeks old) were mated to *W*^*sh*^*/W*^*sh*^ stud males, and fertilized embryos were collected from oviducts. Cas9 mRNAs (from 20 ng/μL to 200 ng/μL) and sgRNAs (from 20 ng/μL to 50 ng/μL), as previously described [19,20], were injected into the cytoplasm of fertilized eggs with well-recognized pronuclei in M2 medium (Sigma). Per previous methods, the injected zygotes were cultured in KSOM with amino acids at 37°C under 5% CO_2_ in air until blastocyst stage by 3.5 days. Thereafter, 15–25 blastocysts were transferred into the uterus of pseudo-pregnant ICR females at 2.5 days post-coitum [19]. Following blastocyst transfer, all pseudo-pregnant females and resulting *W*^*sh*^*/W*^*sh*^ immunocompromised mice were housed in autoclaved, sterile environments.

### Surveyor assay and RFLP analysis for genome modification

The Surveyor assay was performed as described [21]. Genomic DNA from targeted and control mice or blastocysts was extracted and PCR was performed using gene-specific primers (Table 1) under the following conditions: 95°C for 5 min; 35 × (95°C for 30 s, 60°C for 30 s, 68°C for 40 s); 68°C for 2 min; hold at 4°C. PCR products were then denatured, annealed, and treated with Surveyor nuclease (Transgenomics). The DNA concentration of each band was measured on an ethidium-bromide-stained 10% acrylamide Criterion TBE gel (BioRad) and quantified using ImageJ software. For RFLP analysis, 10 μL of *Il2rγ* and 10 μL of *Rag2* PCR product were digested with PvuII. Digested DNA was separated on an ethidium-bromide-stained agarose gel (2%). For sequencing, PCR products were cloned using the Original TA Cloning Kit (Invitrogen), and mutations were identified by Sanger sequencing.

### Flow cytometry

Blood was collected via the submandibular vein using a sterile Goldenrod animal lancet (Medipoint Inc.) and collected into a BD Microtainer MAP containing 1.0mg of K2 EDTA (Becton, Dickinson and Company). Additionally, mice were euthanized with CO_2_ according to institutional guidelines and their spleens were harvested and processed. Single-cell suspensions were generated from the blood and spleen samples and cells were stained using empirically determined concentrations of antibodies for 25 min on ice. Flow cytometry was used to separate T-cells (TCR-Beta, APC-Cy7), B cells (IgM, Alexa Fluor 488), Natural Killer (NK) cells (NK1.1, DsRed) and lymphocytes (FSC/SSC) from either blood or spleen samples. Flow cytometry data analysis was performed using FlowJo software.

### Tumor assay

Human breast carcinoma cell line MCF7 was cultured (a gift from the laboratory of Dr. Robert Weinberg, Whitehead Institute for Biomedical Research) [22] and 1x10^5^ MCF7 cells were resuspended in 250 μL of Dulbecco’s Modified Eagle’s Medium (DMEM; Sigma) containing 10% fetal calf serum (FCS, Sigma). The MCF7 cell suspension was co-injected subcutaneously with 250 μL of matrigel into the flank of NOD SCID gamma mice, *W*^*sh*^*/W*^*sh*^ mice, and *W*^*sh*^*/W*^*sh*^ mice with CRISPR deletions of *Rag2* and *Il2rγ*. Before injection, mice were anesthetized with isoflurane, and allowed to recover to full ambulation immediately after injection. Mice were monitored 2x per day for any signs of adverse health and/or tumor growth. Tumors developed within 6 weeks of injection and animals were euthanized before tumor size exceeded 3 cm in diameter, or inhibited the ability of the mouse to ambulate, following institutional guidelines.

### Necropsy

A total of 15 *W*^*sh*^*/W*^*sh*^ adult mice with knockouts of *Rag2* and *Il2rγ*, comprising 12 female and 3 males from the breeding colony housed in autoclaved cages with autoclaved food, water, and hardwood bedding were submitted for necropsy because of unanticipated increased incidence of morbidity and mortality.

### Culture

At necropsy, brain, blood, kidney, and uterus were harvested for culture using sterile techniques. Tissue samples were plated on chromID™ CPS® agar plates (Biomérieux) [23]. *E*. *coli* colonies growing on CPS or MacConkey agar plates were initially identified by their ability to ferment lactose. Cultures were characterized as *E*. *coli* by API® 20 E (Biomérieux). Feces from an additional 17 immunocompromised mice without clinical signs were cultured for the presence of *pks+ E*. *coli*. Nine rederived mice (6 female and 3 male, *Rag2* and *Il2rγ W*^*sh*^*/W*^*sh*^) were also cultured for *pks+ E*. *coli*.

### DNA extraction and PCR amplification

Thirteen *E*. *coli* isolates were collected with sterile plastic loops placed in sterile PBS in microfuge tubes and used for DNA extraction. The Roche High Pure PCR Template Preparation Kit was used for bacterial DNA extraction. DNA concentration was measured using NanoDrop 2000c (Thermo).

### Serotyping

Four *E*. *coli* isolates from affected mice were submitted to the *E*. *coli* Reference Center at Pennsylvania State University for complete typing which included: O and H typing and analyses and PCR for heat-labile toxin (LT), heat-stabile toxin (*estA* and *estB*), Shiga-type toxin 1 and 2 (*Stx1* and *Stx2*), intimin gamma (*eae*), and cytotoxic necrotizing factor 1 and 2 (*cnf1* and *cnf2*).

### *Clb* genes and phylogenetic group identified by PCR

PCRs were performed to detect *pks (clbA* and *clbQ*) and *cdtB* genes with primers JPN42 and JPN46 for *clbA*, JPN55 and JPN56 for *clbQ*, and cdt1 and cdt2 for *cdtB* gene, respectively [16]. Phylogenetic groups of the isolates were determined with a set of primers published previously [16].

### Cell culture conditions and gentamicin protection assay for colibactin cytotoxicity

*E*. *coli* strains used for cytotoxicity assay included K12 (negative control), NC101 (colibactin positive control), and novel mouse isolates 1512290008 [24], 1512290026 [24], 1601050009, and 1601060011. The cell culture assay for colibactin cytotoxicity was performed as described previously with modifications [5]. HeLa S3 cells (ATCC CCL2.2) were grown and maintained in Eagle's Minimum Essential Medium (EMEM, ATCC) containing fetal calf serum (FCS, Sigma) and 1% Antibiotic-Antimycotic solution (Gibco) at 37°C with 5% CO_2_. Fifty-thousand cells were seeded onto 12-well cell culture plates and incubated at 37°C with 5% CO_2_ for 24 h. Overnight cultures of *E*. *coli* strains were grown for 2 h at 37°C and then adjusted to a multiplicity of infection (MOI, number of bacteria per cell at the onset of infection) of 25 and 100. Following inoculation, plates were centrifuged at 200 g for 10 minutes to facilitate bacteria interaction and then incubated at 37°C with 5% CO_2_ for 4 h. Cells were then washed with EMEM and replaced with EMEM containing 10% fetal calf serum (FCS, Sigma) and 200 μg/mL gentamicin (Gibco). Following 72 h incubation, plates were stained with Diff-quick stain (ThermoScientific). Cells were then inspected under a microscope for confluence and morphological changes. Images were captured with Axiovert-10 microscope (Zeiss) using Image Pro-Plus software version 7.0 at 20x magnification. Cell viability was estimated in the Diff-quick stained plates by quantifying signal intensity at 700 nm using an Odyssey CLx plate reader (LI-COR) and Image Studio version 5.2 software (LI-COR).

### Histopathology and peptic nucleic acid (PNA) fluorescent in situ hybridization

Tissue sections of kidney, heart, lung, brain, spinal cord, ear, and reproductive tract from clinically affected mice were fixed in 10% formalin, embedded in paraffin, sectioned at ~4 μm, and stained with hematoxylin and eosin (H&E). The tissue sections from these clinically affected mice were subjected to PNA fluorescent in situ hybridization by *GNR Traffic Light*^*TM*^ PNA FISH^®^ probe to detect *E*. *coli* [16]. The tissue sections were incubated with the *E*. *coli* specific PNA FISH probe (AdvanDx, Inc., Woburn, MA) for hybridization at 55°C for 105 min and examined for fluorescence with a Zeiss Axioskop 2 plus microscope. Digital images were acquired with a QImaging-QIClick camera (QImaging, Surrey, BC, Canada).

### Rederivation of *Rag2* and *Il2rγ W*^*sh*^*/W*^*sh*^ mice

Mice were rederived by embryo transfer using standard techniques and recipient female Swiss mice free of *pks+ E*. *coli* and other murine pathogens. These mice have been maintained in a separate room under barrier conditions, which consists of maintaining the mice in autoclaved caging with autoclaved bedding, water, and pelleted diet.

### Statistical analyses

Differences in experimental groups were determined by the Student’s t test as appropriate. p values <0.05 were considered significant.

## Results

### CRISPR/Cas9 deletion of *Rag2* and *Il2rγ* in the *W*^*sh*^*/W*^*sh*^ mouse background

**Fig 1:**
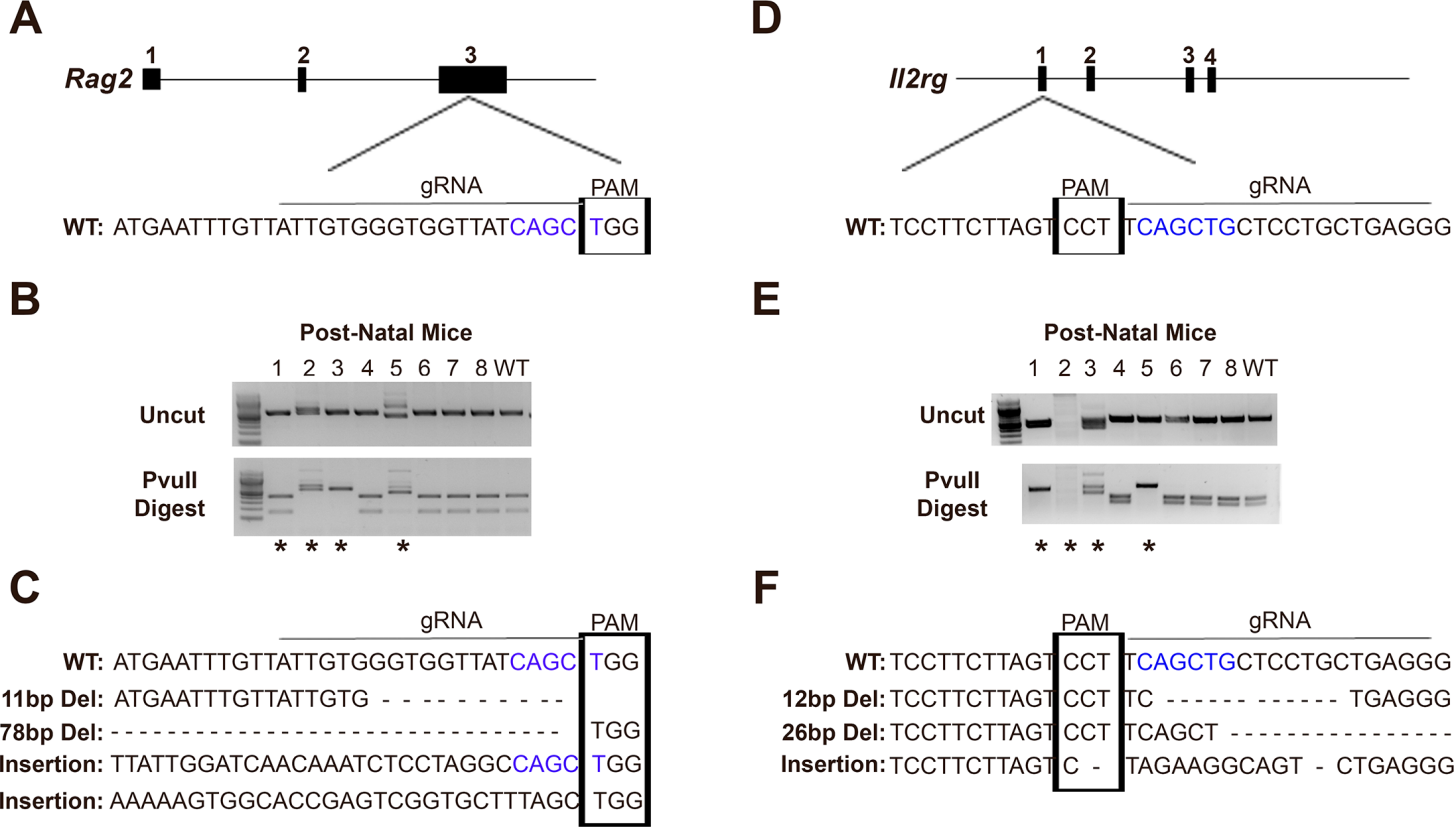
Knockout of *Rag2* and *Il2rγ* in the *W*^*sh*^*/W*^*sh*^ mouse background. (A) Schematic of CRISPR/Cas9 targeting of *Rag2* exon 3. (B) *Rag2* DNA from post-natal mice both uncut (top gel) and cut with PvuII (bottom gel). (C) *Rag2* wild-type (WT) DNA sequence followed by mutations after CRISPR/Cas9 delivery. (D) Schematic of CRISPR/Cas9 targeting of *Il2rγ* exon 1. (E) *Il2rγ* DNA from post-natal mice both uncut (top gel) and cut with PvuII (bottom gel). (F) *Il2rγ* WT DNA sequence followed by mutations found after CRISPR/Cas9 delivery. Straight line above WT genomic sequences, site of guide RNA; Boxed region of genomic sequences, PAM site; Blue font DNA, PvuII cut site; Asterisks, mice chosen as breeders for colony.

Deletion of the host immune response is crucial for survival of human cells after transplantation into murine models, as well as allowing for gene therapy and other clinical approaches to be applied to the animal model without risk of an adverse systemic immune response to the therapy being tested for efficacy. The onset of efficient and rapid genome editing technologies has increased the ability for different mouse strains to become immune-compromised for translational research studies. As an example, CRISPR and Cas proteins have been demonstrated as an efficient gene targeting technology [18, 19]. In our study, capped polyadenylated Cas9 mRNA was produced by *in vitro* transcription and co-injected into pro-nuclear stage one-cell *W*^*sh*^*/W*^*sh*^ mouse embryos with sgRNAs to knockout *Rag2* (Fig 1A) and *Il2r****γ*** (Fig 1D). *Rag2* encodes a protein that is involved in the initiation of V(D)J recombination during B and T cell development while *Il2r****γ*** encodes a protein involved in the growth and maturation of T-cells, B-cells, and NK cells. Therefore, CRISPR/Cas9-induced in-dels or insertions in *Il2r****γ*** and *Rag2* that cause a loss of function of the protein should create an immunocompromised *W*^*sh*^*/W*^*sh*^ mouse strain for further research studies.

RFLP assays on DNA from post-natal mice displayed 3 out of 8 (37.5%) mice had altered *Rag2* alleles (Fig 1B), while 4 out of 8 (50%) mice had altered alleles for *Il2r****γ*** (Fig 1E). Two males (#1: WT for *Rag2* and homozygous mutant for *Il2r****γ***; and #2: homozygous mutant for *Rag2* and unknown, likely a large deletion mutant, for *Il2r****γ***) and two females (#3: homozygous mutant for both *Rag2* and *Il2r****γ***; and #5: homozygous mutant for both *Rag2* and *Il2r****γ***) were chosen for continued breeding to create further generations of *Rag2* and *Il2r****γ*** knockout mice (Fig 1B and 1E, asterisks). Sanger sequencing of the F1 generation displayed four mutant alleles in the population of *Rag2* (Fig 1C) and 3 mutant alleles in the population for *Il2r****γ*** (Fig 1F) that were maintained through generations in the colony.

### Loss of the B, T, and NK cells

**Fig 2:**
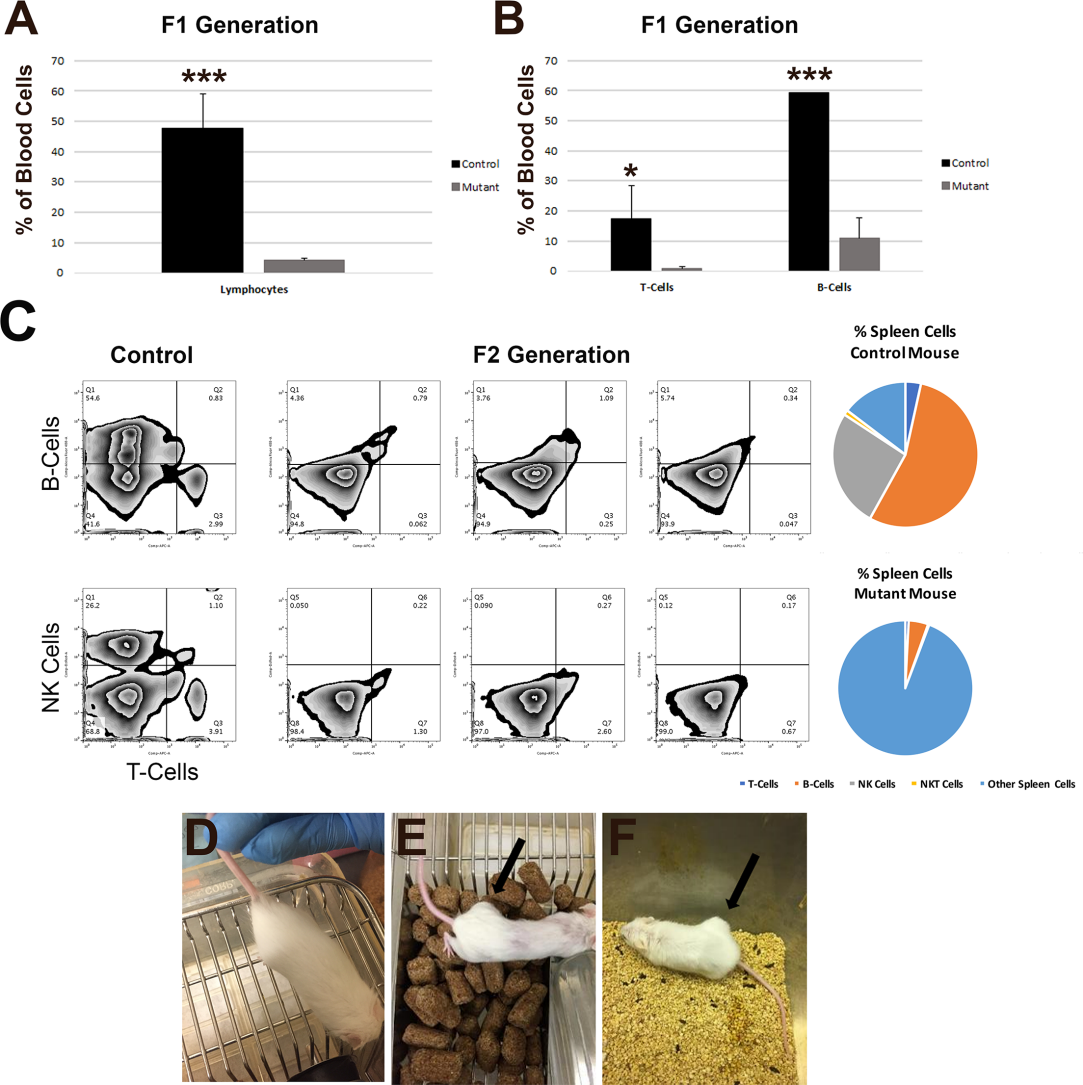
Immunocompromised state of the *Rag2* and *Il2rγ* knockout *W*^*sh*^*/W*^*sh*^ mice. (A) Flow cytometry from blood for lymphocytes in *W*^*sh*^*/W*^*sh*^ control mice and the first (F1) generation of *Rag2* and *Il2rγ* mutant mice. (B) Flow cytometry from blood for the presence of T-cells and B-cells in *W*^*sh*^*/W*^*sh*^ control mice and the F1 generation of *Rag2* and *Il2rγ* mutant mice. *, p < 0.05; *** p < 0.001; N ≥ 2 control mice; N ≥ 4 mutant mice. (C) Flow cytometry of spleen cells in two control (far left) mice compared to three F2 generation *Rag2* and *Il2rγ* mutant mice displaying no detectable populations of B-cells (top row, y-axis), T-cells (top and bottom rows, x-axis), or NK cells (bottom row, y-axis). Percentages of cell types from the flow cytometry analysis are shown for both the control mouse (top right) and mutant moue (bottom right). (D-F) MCF7 human mammary carcinoma cells were injected subcutaneously into the flank of four *W*^*sh*^*/W*^*sh*^ mice (D), an immune-compromised NOD SCID gamma mouse (E), and five *W*^*sh*^*/W*^*sh*^ *Rag2* and *Il2rγ* mutant mice (F). Human MCF7 cells survived and proliferated only in the immunocompromised NOD SCID gamma mouse and *W*^*sh*^*/W*^*sh*^ *Rag2* and *Il2rγ* mutant mice after 6 weeks post-injection. No human cells displayed growth and proliferation in *W*^*sh*^*/W*^*sh*^ mice by 7 weeks post-injection. Arrows, tumor-like growth sites.

Blood samples were collected from *W*^*sh*^*/W*^*sh*^ mice and first generation (F1) *W*^*sh*^*/W*^*sh*^ mice with double allelic mutations and heterozygous mutations in *Rag2* and *Il2r****γ***. As loss-of-function of *Rag2* and *Il2r****γ*** should deplete lymphocytes, and in particular the lymphocyte-derived B-, T-, and NK cells, blood cells were sorted by flow cytometry to quantify the numbers of lymphocytes in the F1 generation compared to control mice (Fig 2A). There was a significant reduction in the number of lymphocytes (47.85% for control mice, 4.30% for mutant mice; p = 0.0002) in the blood from the F1 generation (N ≥ 4) compared to the control mouse cohort (N ≥ 2). Closer examination of the blood to ascertain specific lymphocyte-derived cell types revealed a significant reduction in both T-cells (17.26% for control mice, 0.96% for mutant mice; p = 0.0297) and B-cells (59.29% for control mice, 10.91% for mutant mice; p < 0.0001) in the samples from the F1 generation in comparison to control mice (Fig 2B). NK cells were not present via staining with NK1.1 antibody in any of the F1 generation mice (N = 6; data not shown). Thus, within the first generation of mice, loss of *Rag2* and *Il2r****γ*** even in heterozygotes led to a reduction in the presence of B-, T-, and NK cells.

*W*^*sh*^*/W*^*sh*^ F2 mice carrying double allelic mutations in *Rag2* and *Il2r****γ*** were euthanized and spleen samples were taken for further quantification of immune cells. No lymph nodes were detectable for dissection in any of the F2 generation mice (N = 3; data not shown). Flow cytometry was performed on all three F2 mutant mice compared to two controls, and no visible populations of T-cells, B-cells, NK cells, or NKT cells were detected (Fig 2C). Percentages of each of the B-cells, T-cells, NK cells, NKT-cells, and other, non-immune spleen cells from the flow cytometry analysis are displayed in pie charts for the control mouse (top right) and immune-compromised mutant mouse (bottom right; only a small background of B-cells are visible; Fig 2C). Thus, F2 mice carrying mutations in both alleles of *Rag2* and *Il2r****γ*** have a complete loss of B-, T-, and NK cells as expected with loss-of-function of *Rag2* and *Il2r****γ***.

### A functional immunocompromised state in the *Rag2* and *Il2rγ* knockout *W*^*sh*^*/W*^*sh*^ mice

To ensure that the loss of B-, T-, and NK cells led to a functional immunocompromised state, MCF7 cells were injected subcutaneously into the flank of 5 (3 males and 2 females) *W*^*sh*^*/W*^*sh*^ mice with mutations on both alleles of *Rag2* and *Il2r****γ***. Additionally, MCF7 cells were injected subcutaneously into the flank of 1 NOD SCID gamma mouse, a known immunocompromised mouse model, as well as 4 (2 males and 2 females) *W*^*sh*^*/W*^*sh*^ mice with functional immune systems. Six weeks post-injection, tumors developed in all mice (100%, N = 5) with different *Rag2* and *Il2r****γ*** knockout mutant alleles and a tumor was also noted in the NOD SCID gamma control mouse with a defective immune system which allows proliferation of human mammary carcinoma cells(Fig 2E and 2F, arrows). No tumors formed in any of the *W*^*sh*^*/W*^*sh*^ mice at 7 weeks post-injections, as expected in mice with a functional immune system (Fig 2D). The rejection of MCF7 cells in the parental strain (*W*^*sh*^*/W*^*sh*^ mice) without CRISPR deletion of *Rag2* and *Il2rγ* and proliferation of MCF7 cells in *Rag2* and *Il2rγ* knockout *W*^*sh*^*/W*^*sh*^ mice suggests that these mice are in an immune-compromised state.

### *E*. *coli* isolation from the *Rag2* and *Il2rγ* knockout *W*^*sh*^*/W*^*sh*^ mice

A random survey of the feces from 17 immunocompromised mice being maintained in the colony indicted that 100% of the mice were colonized with *pks+ E*. *coli* (S1 Fig). All 17 pks+ *E*. *coli* isolates belonged to phylogenetic group B2 (S2 Fig).

A sudden increase in morbidity and mortality developed within the *Rag2* and *Il2rγ* knockout *W*^*sh*^*/W*^*sh*^ mice, where mouse colony numbers dramatically declined and it was uncertain whether or not the colony could be maintained. This rapid increase in morbidity and mortality warranted an investigation, as these mice were housed in sterile, autoclaved environments within the barrier animal facility and were not removed at any time. *E*. *coli*, *Enterococcus faecalis*, *Enterococcus faecium*, and *Staphylococcus xylosus* were isolated from multiple tissues and blood of the mice submitted for evaluation. The presence of *E*. *coli* was confirmed in 93% of the cases either by culture or FISH analysis. *Enterococcus* spp. (40%) and *S*. *xylosus* (7%) were concurrently isolated along with *E*. *coli* from these affected mice (Table 2).

**Table 2:** Characterization of *E*. *coli* isolates from *Rag2* and *Il2rγ* knockout *W*^*sh*^*/W*^*sh*^ mice.

Isolate ID	Serotype	Culture Sample	Phylogenetic Group	*clbA*	*clbQ*	*cdtB*	CYT
1512290008	O2:H6	Blood	B2	Positive	Positive	Negative	Positive
1512290026	O2:H6	Blood	B2	Positive	Positive	Negative	Positive
1601050009	O2:H6	Blood	B2	Positive	Positive	Negative	Positive
1601060011	O2:H6	Blood	B2	Positive	Positive	Negative	Positive
1601190003	NT	Uterus	B2	Positive	Positive	Negative	NT
1601190003	NT	Kidney	B2	Positive	Positive	Negative	NT
1601260012	NT	Uterus	B2	Positive	Positive	Negative	NT
1601260012	NT	Kidney	B2	Positive	Positive	Negative	NT
1602030009	NT	Uterus	B2	Positive	Positive	Negative	NT
1606150011	NT	Kidney	B2	Positive	Positive	Negative	NT
1608160086	NT	Kidney	B2	Positive	Positive	Negative	NT
1609230019	NT	Uterus	B2	Positive	Positive	Negative	NT
1609290016	NT	Uterus	B2	Positive	Positive	Negative	NT

CTY, Colibactin cytotoxicity assay; NT, not tested

### Serotyping and analyses of other virulence factors in *E*. *coli*

Four isolates of *E*. *coli* from four mice were determined to be serotype O2:H6 (Table 2). The 4 isolates were positive for *pks (clbA* and *clbQ*) and colibactin cytotoxicity assay. None of these 4 isolates were positive for heat-labile toxin (*elt*), heat-stable enterotoxin a and b (*estA* and *estB*), Shiga-like toxin types 1 and 2 (*stx1* and *stx2*), intimin-*γ* (*eae*), and cytotoxic necrotizing factors 1 and 2 (*cnf1* and *cnf2*).

### Rederivation of *Rag2 and Il2rγ knockout W*^*sh*^*/W*^*sh*^ *mice*

Rederivation of the transgenic mice using embryo transfer produced mice with an intestinal flora devoid of *pks+ E*. *coli*. Six adult female and 3 adult male *Rag2* and *Il2rγ W*^*sh*^*/W*^*sh*^ mice were cultured for the presence of *pks+ E*. *coli* strains using standard culture methods. Importantly, these barrier-maintained rederived mice have produced multiple litters without adverse health effects.

### *Clb* genes and phylogenetic group identified by PCR

**Fig 3:**
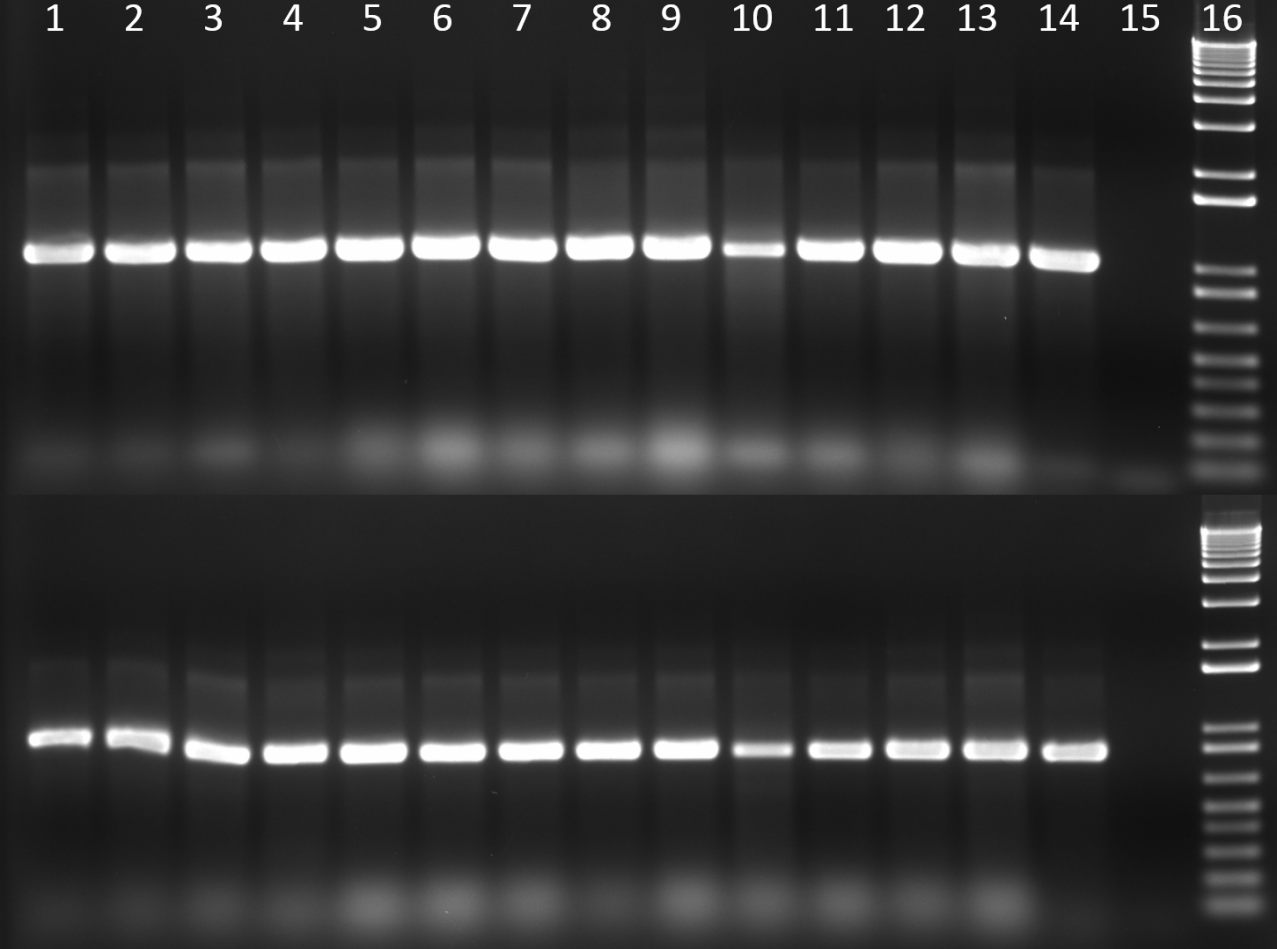
Amplification of *clbA* and *clbQ* in DNA from mouse *E*. *coli* isolates. Top row: *clbA* gene, bottom row: *clbQ* gene. Lane 1 to lane 13, 13 *E*. *coli* isolates from mice samples; line 14, NC101 (positive control); line 15, no DNA control; line 16, 1 Kb plus molecular marker.

**Fig 4:**
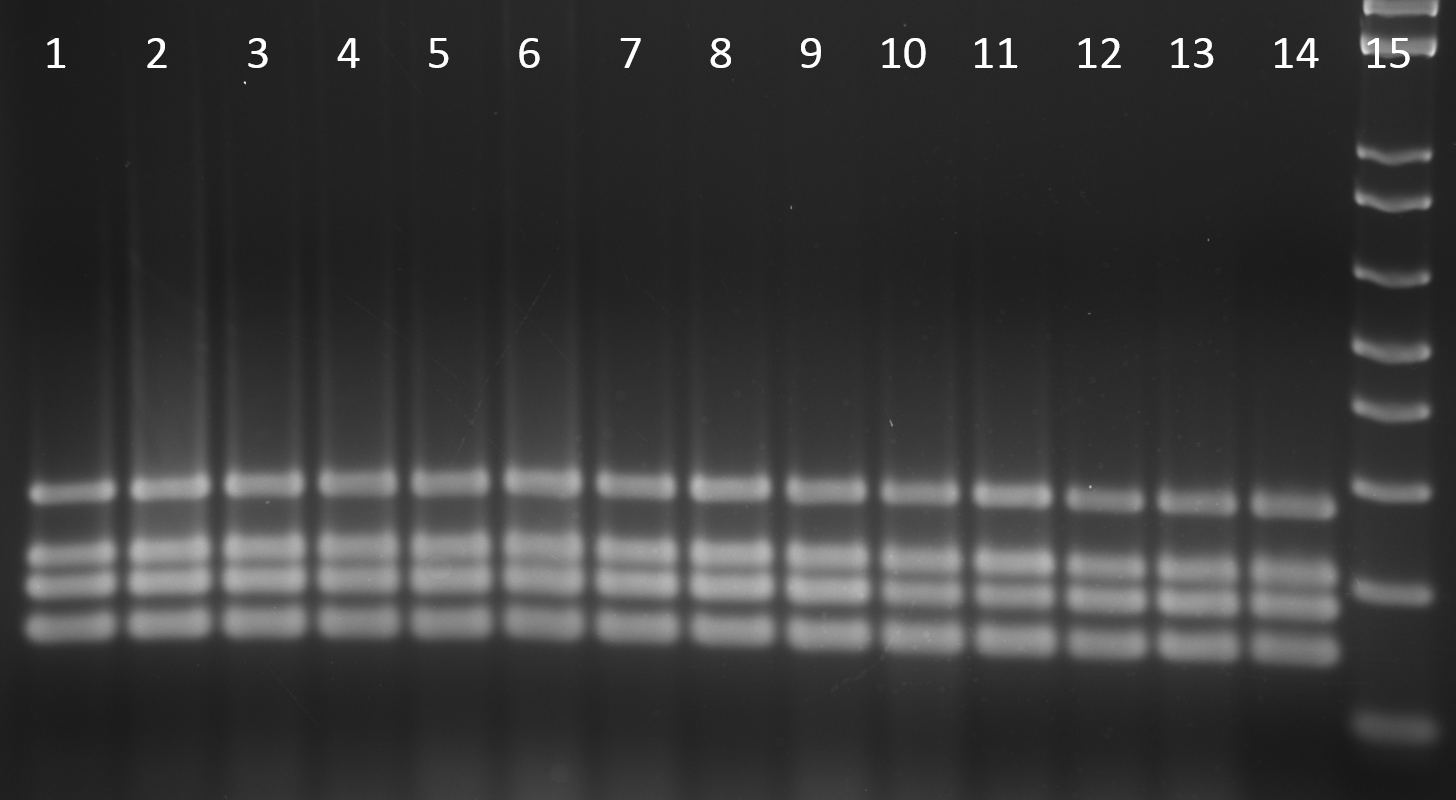
Phylogenetic group determination of *E*. *coli*. Lane 1 to lane 13, 13 *E*. *coli* isolates from mice samples; line 14, NC101 (positive control); line 15, 1 Kb plus molecular marker. All samples were determined to be phylogenetic group B2.

Thirteen *E*. *coli* strains from ten mice were positive for *clbA* and *clbQ* genes and negative for *cdtB* (Table 2 and Fig 3). All isolates belong in phylogenetic group B2 (Table 2 and Fig 4).

### *Clb*-encoding mouse *E*. *coli* isolates exert colibactin cytotoxicity

**Fig 5:**
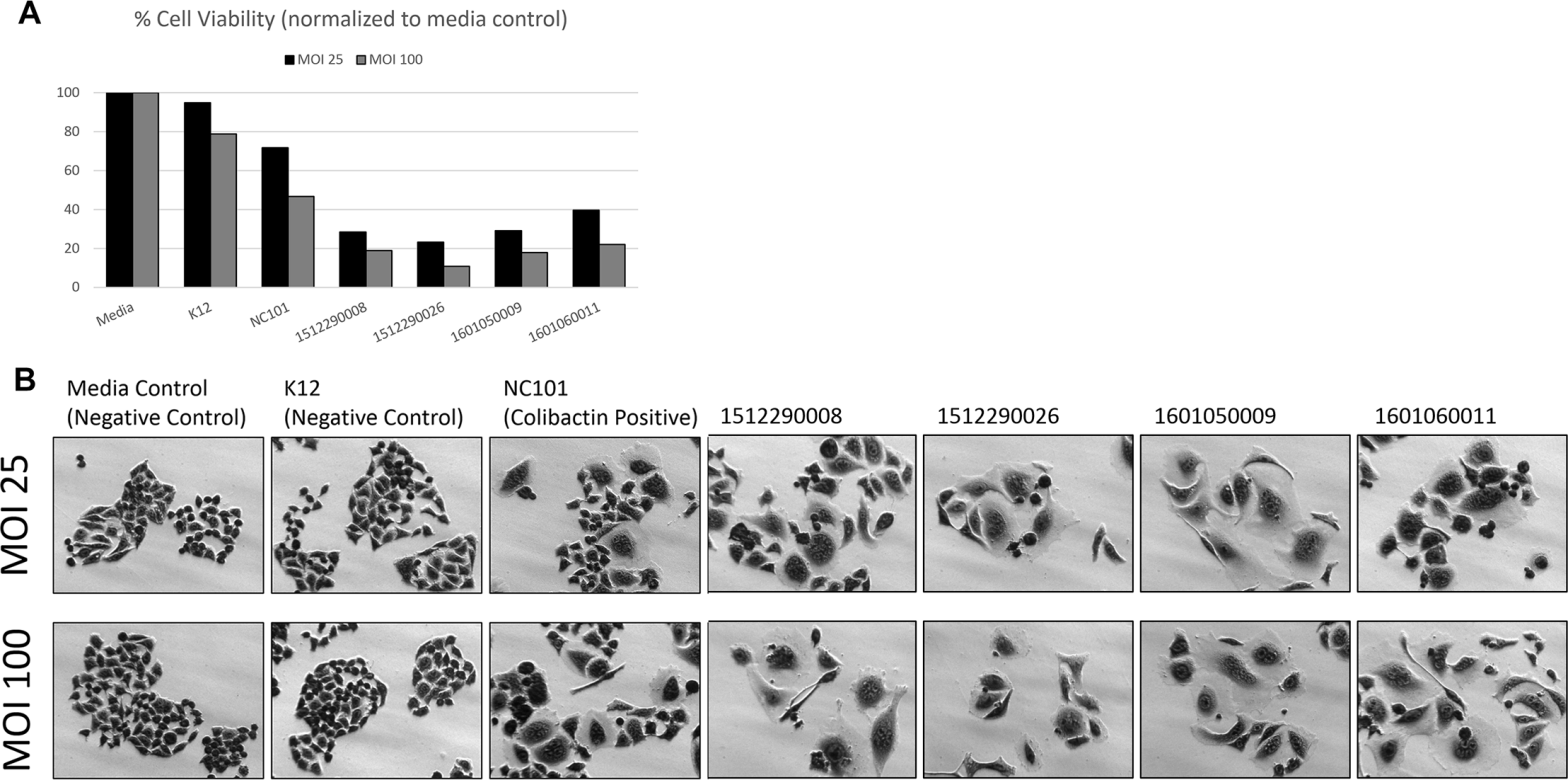
HeLa cell colibactin cytotoxicity assay. HeLa cells were inoculated with *E*. *coli* at a multiplicity of infection (MOI) of 25 and 100 for 4 h followed by a 72 h incubation in gentamicin-containing media. Megalocytosis (enlargement of the cell body and nucleus) was observed in cells infected with the colibactin (Clb)-encoding novel mouse *E*. *coli* isolates 1512290008, 1512290026, 1601050009, and 1601060011. NC101 is a Clb-encoding mouse *E*. *coli* isolate (positive control). K12 is a non-pathogenic *E*. *coli* strain (negative control). A) Estimation of cell viability. B) Representative images were taken at 20X magnification.

To confirm cytotoxic colibactin activity in the clb-encoding novel mouse *E*. *coli* isolates, HeLa cells were transiently infected with live bacteria at MOI 25 and 100. Novel mouse isolates 1512290008, 1512290026, 1601050009, and 1601060011 induced dose-dependent cytotoxicity. About 30–40% of cells survived after MOI 25, while less than 20% survived after MOI 100 (Fig 5A). Surviving cells appeared megalocytic and were phenotypically indistinguishable to that caused by Clb-encoding NC101 infection (Fig 5B). Media and K12 negative controls maintained similar cell confluence and morphology (Fig 5B).

### Histopathological evaluation

A total of fifteen *Rag2* and *Il2rγ* knockout *W*^*sh*^*/W*^*sh*^ mice comprising 12 females and 3 males were submitted for post mortem examination. Both males and females became moribund during our study, usually before 1 month of age; however, breeding females became ill at a rapid rate, either during the final week of pregnancy or within 1 week of nursing pups, and therefore more severely ill female mice were available for analysis. Table 3 summarizes the major organ systems affected, nature of the lesion and bacteria isolated and identified in different organ systems by microbial culture and FISH assay. The three affected male mice had pale tan shrunken kidneys with irregularly pitted capsular surface. Histological changes in the kidneys from the affected male mice were consistent with severe necrotizing granulocytic pyelonephritis with abundant bacteria and tubular degeneration and necrosis. Further characterization was performed on tissues/organs from the 12 female mice, to avoid any confounding factors related to gender.

**Table 3:** Pathology of spontaneous infection by *E*. *coli* in *Rag2* and *Il2rγ* knockout *W*^*sh*^*/W*^*sh*^ mice.

Accession #	Sex	Microbial Culture				Histologic lesions	FISH- *E*. *coli*
		*E*. *coli*	*E*. *faecalis*	*E*. *faecium*	*S*. *xylosus*		
1512290008	Male	bl,n,lu,s,k	l	n		Pyelonephritis and interstitial pneumonia	ND
1512290026	Male	bl,n,lu,k	bl			Pyelonephritis	Kidney
1601050009	Female	bl				Metritis with macerated fetus, pyelonephritis, pneumonia, otitis media (bilateral), and epicarditis	Uterus and placenta
1601190003	Female	lu, li, k,u				Placentitis, metritis, and pneumonia	Uterus and placenta
1601260012	Female	lu,li,k,u	lu,k,u			Metritis, vaginitis myocarditis, meningoencephalitis, otitis media(unilateral), and pneumonia	Kidneys, uterus, vagina, and heart
1602030009	Female	lu,u	lu			Metritis, vaginitis, myocarditis, meningoencephalomyelitis, necrotizing hepatitis, unilateral otitis media and rhinitis	Uterus and placenta
1606150011	Male	k, br,bl	k, br	br		Nephropathy (intralesional bacteria)	Kidneys
1608160086	Female	k				Metritis and necrotizing suppurative pyelonephritis	Kidneys
1609230019	Female	u				Metritis, nephritis, interstitial pneumonia, and meningitis	Uterus
1609290016	Female	k	k,u		k,u	Pyelonephritis and interstitial pneumonia	ND
ND16-00081	Female	No bacterial isolation was performed, as the animals were found dead				Metritis with abundant intralesional bacteria and macerated fetus and pneumonia	Uterus and placenta
ND16-00082	Female					Cystitis and vaginitis with intralesional bacilli	Bladder and urogenital tract
ND16-00083	Female					Metritis and unilateral otitis media with intrahistiocytic bacteria	Uterus
ND16-00094	Female					Endometritis with macerated fetus and unilateral otitis media	ND
ND16-00164	Female					Pyelonephritis cystitis, endometritis, bilateral otitis media, rhinitis with intralesional bacteria, interstitial pneumonia, and meningitis	Uterus

blood-bl, brain-br, kidneys-k, lungs-lu, liver-li, nares-n, spleen-s and uterus-u and ND: Not detected.

**Fig 6:**
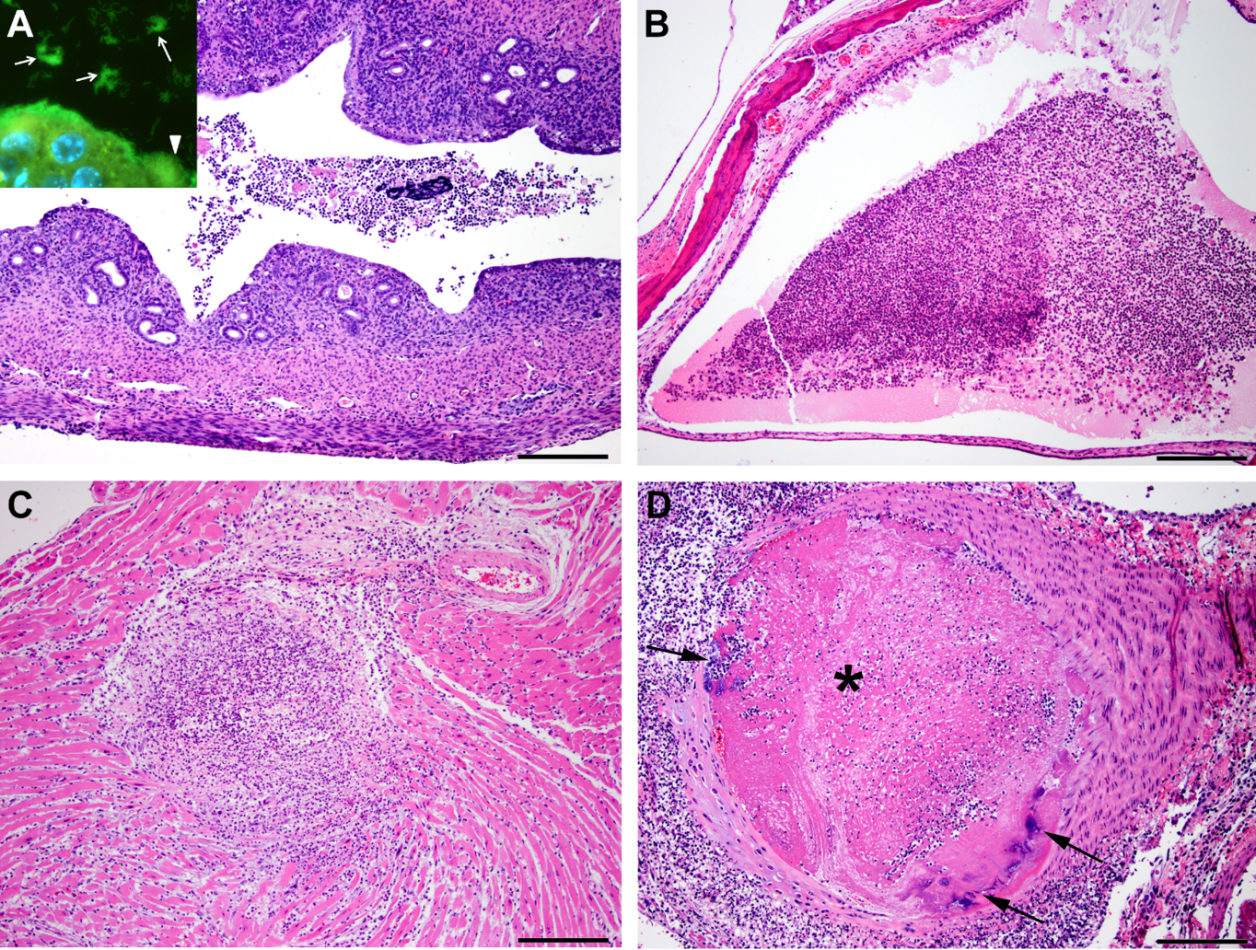
**Histopathological lesions induced by *pks+ E*. *coli*** (A) Mild subacute endometritis. The endometrium is disrupted by low numbers of inflammatory cells and ectatic glands that rarely contain pale eosinophilic material. The uterine lumen contains cellular debris and degenerate inflammatory cells. H&E Scale: 200μm. *Inset*: PNA-FISH assay with *E*. *coli* specific probe reveals colonies of fluorescent green *E*. *coli* bacteria lining the endometrium (white arrow head) and within the uterine lumen (white arrows). (B) Moderate focally extensive subacute otitis media. The middle ear lumen contains moderate amounts of pale esoinophilic fibrillar material, cellular debris, degenerate granulocytes, and mononuclear cells. H&E Scale: 200μm. (C) Moderate focally extensive subacute necrotizing myocarditis. The myocardium was focally disrupted by granulocytic and mononuclear cell infiltration, necrosis, and degeneration of the cardiomyocytes. H&E Scale: 200μm. (D) Aorta: Severe subacute necrotizing valvulitis and arteritis with fibrin thrombi (asterisk) and colonies of bacteria (black arrows). The aortic valve is focally disrupted by necrosis, few bacterial colonies, granulocytes, and mononuclear cell infiltrates.The aortic lumen is occluded by thrombus composed of fibrin, inflammatory cells, and bacteria. H&E Scale: 200μm.

On gross examination, 4 of the 12 female mice were diagnosed with vaginal septum and pyometra. The vaginal orifice of the affected female mice was longitudinally bisected by a thin dorsoventral band of tissue. The urogenital tract and uterus contained pale tan to yellow, turbid, viscous material. Histopathological examination of the uterus and urogenital tract of 11 female mice revealed intraluminal cellular debris admixed with degenerate granulocytes, mononuclear cells, and myriad of bacteria (Fig 6A). The endometrium and underlying myometrium were also infiltrated by inflammatory aggregates, in a multifocal pattern.

The ear canal was affected by mild to moderate unilateral or bilateral otitis media in 6 out of 12 female mice. The otitis media was characterized by loss of ciliated epithelial lining and the lumen contained cellular debris, pale eosinophilic material, and low numbers of granulocytes and histiocytes admixed with extracellular and intrahistiocytic bacilli (Fig 6B).

The heart was affected by necrotizing granulocytic myocarditis (Fig 6C) and epicarditis with mural septic thrombi in 3 out of 12 female mice. The aortic valve was focally disrupted by necrotizing valvulitis characterized by loss of fibrous connective tissue and cartilage, with granulocytic and mononuclear cell infiltrates admixed with bacterial colonies. The aorta was completely occluded by septic thrombi and transmurally, the aortic wall was infiltrated by granulocytes and mononuclear cells (Fig 6D).

**Fig 7:**
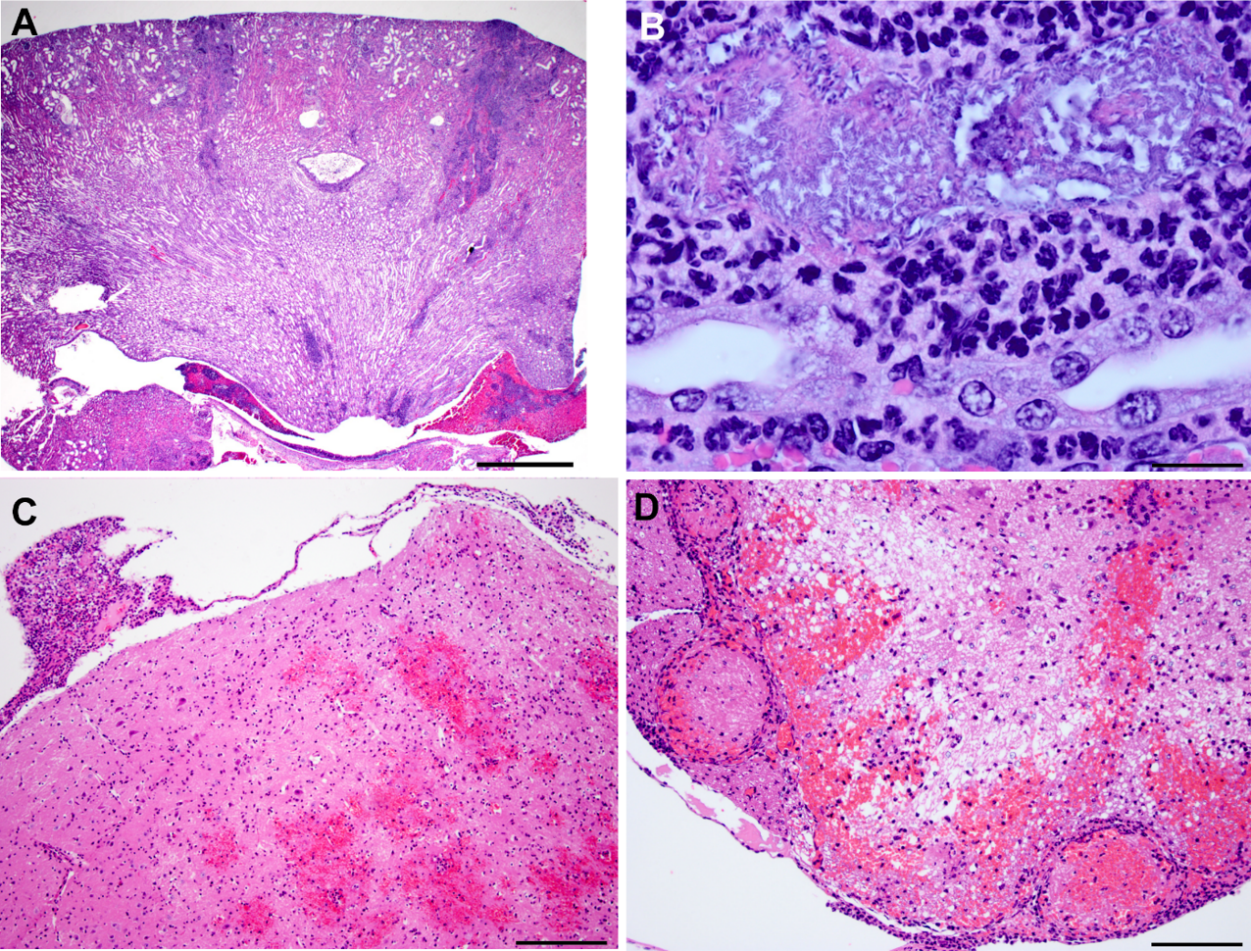
**Histopathological lesions induced by *pks+ E*. *coli*** (A) Moderate multifocal subacute suppurative pyelonephritis with tubular necrosis and intraluminal bacterial colonies. The renal architecture is multifocally disrupted by pyelonephritis that discretely extends from the capsular surface deep in to the medulla. The ectatic renal pelvis contains hemorrhage admixed with inflammatory cells. H&E Scale: 1mm. (B) Higher magnification of the affected renal tubule. Focally, ectatic tubule contained large numbers of bacteria admixed with cellular debris. The epithelial lining is completely lost with intact basement membrane. The adjacent tubular interstitium is infiltrated by granulocytes and mononuclear cells. H&E Scale: 20μm. (C) Mild focally extensive subacute necrohemorrhagic meningoencephalitis. The brain parenchyma and meninges are multifocally disrupted by hemorrhage and inflammatory infiltrates. H&E Scale: 200μm. (D) Lumbar spinal cord: Moderate multifocal subacute hemorrhagic meningiomyelitis with vasculitis and fibrin thrombi. The gray and white matter are disrupted by hemorrhage, vacuolations, granulocytes, and mononuclear cells. The meningeal blood vessels are multifocally affected by vasculitis and fibrin thrombi.

In 5 out of 12 female mice, the renal architecture was multifocally disrupted by necrosis and inflammation. The renal pelvis was dilated and contained hemorrhage and inflammatory cells (Fig 7A). The affected renal tubules were lined by attenuated epithelium and occasionally the tubular epithelial cells with intact basement membrane were lost. The tubular lumen contained cellular debris and abundant bacteria. The tubular interstitium was multifocally infiltrated by granulocytes and mononuclear cells (Fig 7B).

In 4 out of 12 female mice, the brain and spinal cord were variably affected by necrohemorrhagic meningoencephalomyelitis. The affected brain and spinal cord parenchyma were disrupted by minimal amounts of hemorrhage and vacuolations. These affected areas were infiltrated by granulocytes and mononuclear infiltrates and the meningeal blood vessels were disrupted by necrotizing vasculitis and fibrin thrombi (Fig 7C and 7D). Other significant findings in these affected mice included interstitial pneumonia, rhinitis, necrotizing granulocytic hepatitis, and mild granulocytic ureteritis and cystitis.

Since all of the above mentioned organs showed significant findings and signs of disease, we hypothesized that these tissues may have been infected with *E*. *coli*. Thus, representative tissue sections from the uterus, kidneys, urogenital tract, heart, and ear were subjected to *E*. *coli* specific PNA-FISH assay [16]. The PNA-FISH assay revealed *E*. *coli* positivity by the presence of multifocal and discrete clumps of intra luminal bacilli in the tissue sections from the above mentioned organs (Fig 6A).

Further, four rederived mice (3 males and 1 female), confirmed negative for *pks+ E*. *coli*, were analyzed by a detailed gross necropsy followed by histopathological evaluation of the liver, spleen, pancreas, mesenteric lymph node, kidneys, heart, lungs, urinary bladder, reproductive organs (testes, epididymis, prostate or uterus, ovaries, vagina and vulva). No significant pathological findings were noted other than incidental findings of sparse to minimal perivascular interstitial lympho-plasmacytic inflammatory aggregates in the all the renal medulla and/or pelvis of all animals. The female mouse had a gravid uterus.

## Discussion

Although *E*. *coli* infections in laboratory mice have been previously reported [10,13,25–29], a description of spontaneous disease which includes biochemical, genetic, and phenotypic characterization of *pks+ E*. *coli* isolates has not been undertaken. In one of the previously reported studies, natural infection with uncharacterized *E*. *coli* was associated with urogenital lesions, pneumonia, and septicemia in mice [26]. Characterization of *pks+ E*. *coli* NC101 in experimentally infected IL10^-/-^ mice and the natural and experimental characterization of O7:H7:K1 in IL10^-/-^ mice indicated the pathogenic potential of Clb-encoding *E*. *coli* in experimentally infected susceptible animals [10,11,13,30]. The current study characterized *pks+ E*. *coli* in clinically affected mice with genitourinary infection, septicemia, and meningitis. The mouse *E*. *coli* isolates encoding two Clb genes (*clbQ* and *clbA*), were cytotoxic to cells *in vitro*. The isolates in this study were serotype O2:H6, which is the same serotype as that of NC101 [12]. Serotype O2 in humans is also commonly associated with UTIs, septicemia, and meningitis [31]. Importantly, barrier-maintained rederived mice without *pks+ E*. *coli* in their intestinal tracts have produced multiple litters without adverse health effects, nor have lesions noted in the original outbreak observed.

The isolates in the current study were cytotoxic to cells *in vitro* suggesting that mouse Clb-encoding *E*. *coli* have the potential to induce clinical disease in laboratory animals, as noted in this study. In a rodent model of septicemia, 8–9 week old female C57BL/6J mice were injected subcutaneously with a *pks+ E*. *coli* colibactin producing 018:K1:H7, ExPEC strain S15 isolated from a meningitis case in a newborn infant [14]. The *E*. *coli* induced a profound lymphopenia in septicemic mice, which was attenuated when an isogenic mutant lacking colibactin genotoxin activity was injected into the mice [14]. The authors argued that the production of colibactin by *E*. *coli* exacerbates lymphopenia associated with septicemia, reducing the chances that the mice, and by association humans, would survive *pks+ E*. *coli* induced sepsis [14]. The prolonged immunosuppression often referred to as immunoparalysis, in sepsis cases, predisposes the patients to nosocomial infections and reactivation of latent viruses [32,33]. Isolation of commensals such as *Enterococcus* spp. and *S*. *xylosus* along with *pks+ E*. *coli* from these affected mice in our study may be attributed to immunosuppression. *E*. *faecalis* has been shown to exacerbate pathogenic effects induced by Gram-negative bacterial infection [34]. However, *E*. *coli* was isolated in 93% of cases in this study either with or without concurrent isolation of *Enterococcus* spp. and *S*. *xylosus*. Recent studies have demonstrated the importance of colibactin in intestinal colonization and invasion of other organs by *pks+ E*. *coli* that leads to systemic infection in neonates [15]. Hence, there is a distinct possibility that *pks+ E*. *coli* infection facilitates colonization and/or co-infection by other commensal bacteria such as *Enterococcus* spp. and *S*. *xylosus*. Furthermore, our findings of *pks+ E*. *coli* strains responsible for urosepsis in C57BL/6 *Rag2* and *Il2rγ* knockout *W*^*sh*^*/W*^*sh*^ mice reinforce the susceptibility of immuno-compromised mice to opportunistic pathogens.

The breeding colony of *Rag2* and *Il2rγ* knockout *W*^*sh*^*/W*^*sh*^ mice also presented with a variable incidence of vaginal septa. The vaginal septal defect apparently has not been noted to interfere in the overall breeding performance in this colony. Our results however, suggest that the vaginal defect may have predisposed the female mice to *pks+ E*. *coli* urosepsis. The congenital vaginal septal defect has been reported previously and has been associated with compromising successful breeding and parturition, in both mice and rats [35,36]. The vaginal defect has been linked to metritis in rats used on reproductive toxicology studies [36]. Although rats with vaginal septal defects were screened for rat microbial pathogens, none were noted. However, the authors mention that bacterial cultures obtained from abnormal uterine contents identified the presence of abundant *E*. *coli*. Unfortunately, further molecular identification of putative virulence factors was not undertaken [36].

Investigators have also capitalized on the use of neonatal rats to explore the pathogenicity of colibactin expressing *E*. *coli*. Two day old Wistar rats were orally dosed with *pks+ E*. *coli* which efficiently colonized the intestinal tract and translocated across the immature GI tract resulting in sepsis [15]. Inactivation of the *clbA* and *clbP* genes responsible for colibactin production in *pks+ E*. *coli* strains, significantly reduced the capacity of the *E*. *coli* strain A192PP to colonize the intestine, translocate, induce septicemia, and cause the death of the neonatal rats [15]. This neonatal rat model replicates the age dependency seen in newborn children where ExPEC B2 strains of *pks+ E*. *coli* are noted to express colibactin and cause significant disease [37]. Indeed, ExPEC infections are associated with urosepsis, bacteremia, and neonatal meningitis in infants [15,38,39]. *E*. *coli* isolates from human prostatitis cases also have been characterized as ExPEC and some encode Clb [40].

Previously, we reported the draft genomes of two *E*. *coli* isolates from this study that were recovered from the blood of mice (accession numbers 1512290008 and 1512290026)[24]. Both genomes contained a complete ~54-kb PKS pathogenicity island required for colibactin synthesis[24]. Cytolethal distending toxin (*cdt*), cytotoxic necrotizing factor (*cnf*), and cycle inhibiting factor (*cif*) genes known to cause megalocytosis were not present in either of these genomes, which substantiated our PCR results, suggesting the megalocytic cytotoxicity observed in our *in vitro* infection experiments was the result of colibactin activity. Aside from PKS, both of our novel *pks+ E*. *coli* isolate genomes also contained homologous virulence genes that regulate colonization, adherence, immune evasion, cytotoxicity, and iron acquisition at intestinal, vascular, and renal sites by EPEC and ExPEC strains. These genes included enterobactin siderophore receptor protein[41,42], s-fimbriae minor subunit[43], glutamate decarboxylase[44,45], per-activated serine protease autotransporter enterotoxin[46–48], and iss/bor protein precursor[49,50]. The genomic presence of *pks* and other virulence genes supports our hypothesis that these novel mouse *E*. *coli* isolates have the pathogenic potential to infect and induce clinical diseases in susceptible hosts.

The genetic profile of the *Rag2* and *Il2rγ* knockout *W*^*sh*^*/W*^*sh*^ mice predisposes them to progressive kidney disease. The underlying mutation in the *W*^*sh*^*/W*^*sh*^ strains is an inversion mutation in the upstream transcriptional regulatory elements of the c-kit gene that also disrupts the corin gene [51–53]. Corin is a trypsin-like transmembrane serine protease that activates atrial natriuretic peptide, which regulates salt-water balance and blood pressure by promoting renal natriuresis. In humans, abnormalities in corin expression result in hypertension [54], heart failure [55], and chronic kidney disease [56]. In our study, the tubular lesions noted in the mice closely resemble obstructive nephropathy. However, there was no evidence for obstruction of renal tubules, urinary bladder, and urethra by crystals, proteinaceous plugs, and associated inflammation. The observation from our study and findings reported in previous studies suggests decreased expression of c-kit and corin could have resulted in the chronic progressive nephropathy observed in these mice due to impaired sodium excretion. However, the exact mechanism by which c-kit and corin exert this detrimental effect on murine renal tubular epithelial cells and whether this defect predisposes the mice to urosepsis remains to be elucidated.

In summary, the pks pathogenicity island is present in a high percentage of extraintestinal pathogenic B2 strains of *E*. *coli* and exhibits an increased predilection of causing bacteremia [3]. This current finding of *pks+ E*. *coli* strains in cases of urosepsis, as well as *pks*+ *E*. *coli* in the feces of clinically normal *Rag2* and *Il2rγ*^-/-^ mice, and our recent characterization of *E*. *coli* strains from clinically normal and clinically affected laboratory mice indicates that mice can be colonized with Clb-encoding cytotoxic and pathogenic *E*. *coli* [16]. Phylogenetic B2 *pks+ E*. *coli* strains have a high likelihood of persistently colonizing infants and are commonly isolated in adults. Given that *pks+ E*. *coli* B2 isolates are present within 6-12-month-old mice obtained from a mouse colony endemically infected with *pks+ E*. *coli*, this supports the hypothesis that mice can also be persistently infected with this colibactin-producing *E*. *coli* [16]. The epidemiology and virulence determinants of mouse *pks+ E*. *coli* strains should be investigated further. Also, studies examining their impact in mouse models of inflammation, both intestinal and extraintestinal should be conducted. Importantly, our findings of urosepsis in immunocompromised mice caused by *pks+ E*. *coli* strain O2:H6 represents an opportunity to develop experimental mouse models to investigate the pathogenic potential of *pks+ E*. *coli* and cytotoxin-associated induction of urogenital disease and septicemia noted in humans.

Many murine models used in human research are genetically engineered to be immune-compromised in order to test human cell transplantations and viral gene therapies for translational research. This report is the first to describe acute morbidity and mortality associated with *pks+ E*. *coli* urosepsis and meningitis in immunocompromised mice. As *E*. *coli* strains have been frequently isolated from rodents, but are not routinely or completely characterized, our study highlights the importance of routine and complete characterization of *pks+ E*. *coli*. Also, health surveillance strategies to ensure that these *E*. *coli* strains are excluded from specific pathogen-free mouse colonies should be instituted.

## Supporting information

S1 Fig Amplification of *clb*A and *clb*Q in DNA from 17 mouse *E*.*coli* isolates. Top row: *clb*A gene, bottom row: *clb*Q gene. Lane 1 to lane 17, 17 E.coli isolates from mice fecal samples; line 18, NC101 (positive control); line 19, no DNA control; line 20, 1 Kb plus molecular marker.
(PDF)

S2 Fig Phylogenetic group determination of *E*.*coli*. Lane 1 to lane 17, 17 E.coli isolates from mice fecal samples; line 18, NC101 (B2 group positive control); line 19, no DNA control; line 20, 1 Kb plus molecular marker.
(PDF)
