# Supplementary material for: Cytotoxic Escherichia coli strains encoding colibactin isolated from immunocompromised mice with urosepsis and meningitis
Source: PLoS One. 2018 Mar 19;13(3):e0194443. doi: 10.1371/journal.pone.0194443 (PMC5858775; doi:10.1371/journal.pone.0194443)
Supplement: S1 Fig — Top row: clbA gene, bottom row: clbQ gene. Lane 1 to lane 17, 17 E.coli isolates from mice fecal samples; line 18, NC101 (positive control); line 19, no DNA control; line 20, 1 Kb plus molecular marker. (PDF) [file pone.0194443.s001.pdf]

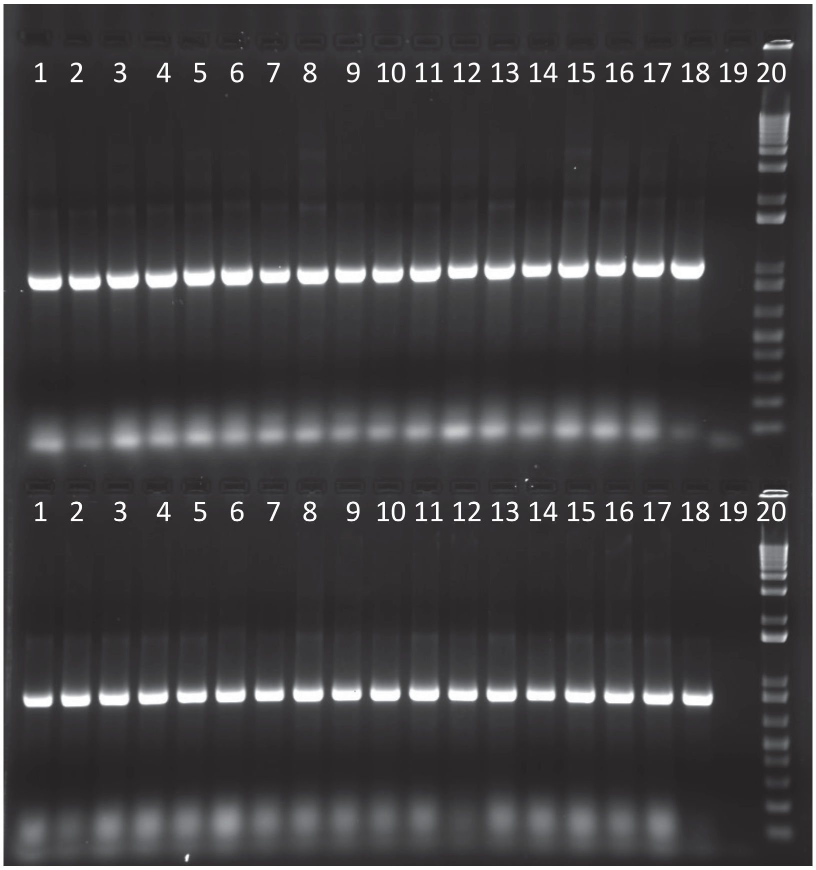

S1 Fig. Amplification of *clb*A and *clb*Q in DNA from 17 mouse *E.coli* isolates. Top row*:* *clb*A gene, bottom row: *clb*Q gene. Lane 1 to lane 17, 17 E.coli isolates from mice fecal samples; line 18, NC101 (positive control); line 19, no DNA control; line 20, 1 Kb plus molecular marker.
