# Supplementary material for: Cytotoxic Escherichia coli strains encoding colibactin isolated from immunocompromised mice with urosepsis and meningitis
Source: PLoS One. 2018 Mar 19;13(3):e0194443. doi: 10.1371/journal.pone.0194443 (PMC5858775; doi:10.1371/journal.pone.0194443)
Supplement: S2 Fig — Lane 1 to lane 17, 17 E.coli isolates from mice fecal samples; line 18, NC101 (B2 group positive control); line 19, no DNA control; line 20, 1 Kb plus molecular marker. (PDF) [file pone.0194443.s002.pdf]

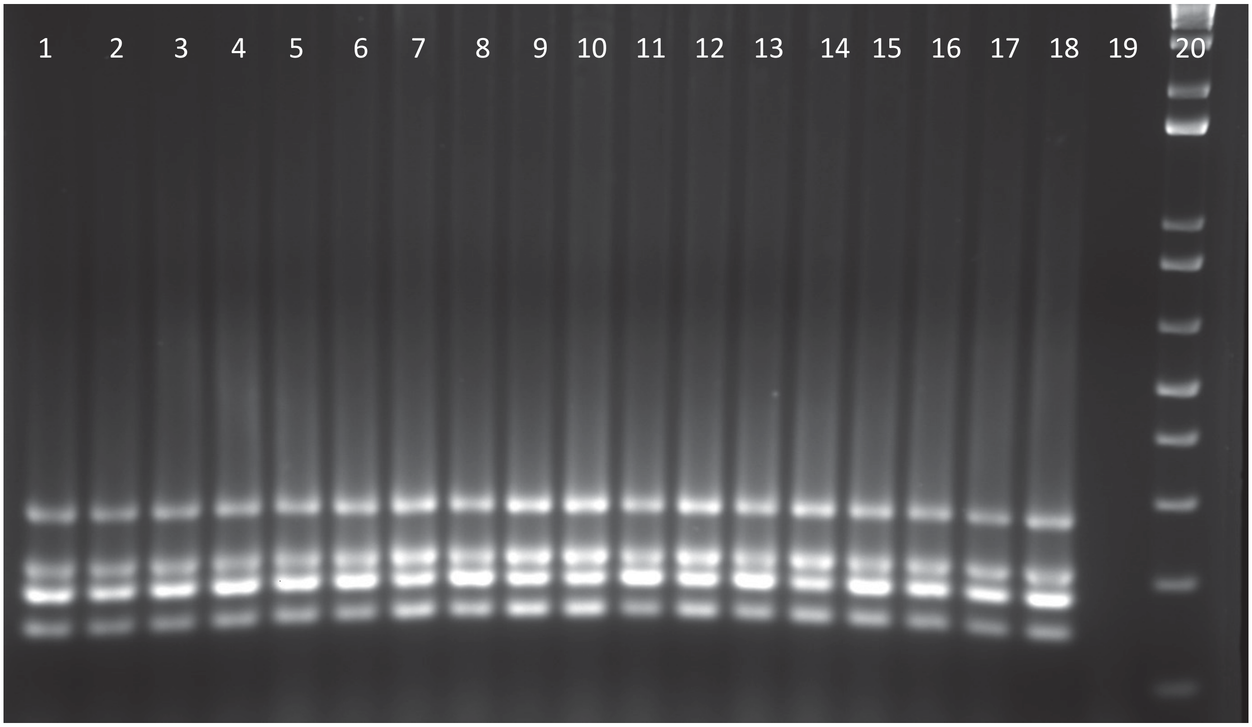


**S2 Fig. Phylogenetic group determination of *E.coli*.** Lane 1 to lane 17, 17 E.coli isolates from mice fecal samples; line 18, NC101 (B2 group positive control); line 19, no DNA control; line 20, 1 Kb plus molecular marker.
